# Nutritional characterization of freshwater mud eel (*Monopterus cuchia*) muscle cooked by different thermal processes

**DOI:** 10.1002/fsn3.1920

**Published:** 2020-10-05

**Authors:** Md. Aminur Islam, Md. Mohibbullah, Sharmin Suraiya, Md. Sarower‐E‐Mahfuj, Shafi Ahmed, Monjurul Haq

**Affiliations:** ^1^ Department of Nutrition and Food Technology Jashore University of Science and Technology Jashore Bangladesh; ^2^ Department of Fisheries and Marine Bioscience Jashore University of Science and Technology Jashore Bangladesh; ^3^ Department of Fisheries and Marine Bioscience Bangabandhu Sheikh Mujibur Rahman Science and Technology University Gopalganj Bangladesh; ^4^ Department of Agro Product Processing Technology Jashore University of Science and Technology Jashore Bangladesh

**Keywords:** amino acids, cooking process, fatty acids, freshwater mud eel, health risk assessments, minerals and heavy metals

## Abstract

This paper reports the effects of four popular cooking methods viz. grilling, boiling, frying, and microwaving on the proximate and nutritional compositions of freshwater mud eel (FWME) muscle. The moisture content of raw FWME muscle was 74.45%, which was similar in boiled products but lower in grilled, fried, and microwaved products (*p* ≤ .05). The protein content in raw and cooked FWME muscles varied between 14.49% and 21.28%. There were found 20 different fatty acids in FWME muscle of which palmitic acid was the most abundant one with an amount of 26.51%–29.70% in raw and cooked FWME muscles. FWME muscle contained a substantial amount of ω‐3 polyunsaturated fatty acids, ranging from 7.54% to 13.7%. However, the thermal effects during cooking decreased the ω‐3 polyunsaturated fatty acid contents. There were seven essential and eight nonessential amino acids available in FWME muscle; among the essential amino acids, lysine content was the highest. Raw and cooked FWME were very rich in calcium, between 794.52 mg/100 g and 883.24 mg/100 g muscle. Among the studied heavy metals, Pb content was the highest. However, all the heavy metal contents were within acceptable limits determined by health risk assessment, that is, target hazard quotient and target cancer risk.

## Graphical abstract

Freshwater mud eel muscle was cooked by four different thermal treatment. The nutritional properties of different cooked products were evaluated.

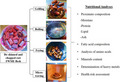

## 1 INTRODUCTION

Freshwater mud eel (FWME) (*Monopterus cuchia*), belonging to the family synbranchidae of the order synbranchiformes, is commonly available in freshwater bodies such as ponds, rivers, haors, baors, canals, beels, and flood plains of Southeast Asian countries including Bangladesh, India, and Pakistan (Jingran & Talwar, 1991). Among different FWME species available in Bangladesh, *Monopterus cuchia* is very common; it is one of the most important fish species of Bangladesh with high export demand (Islam, 2017). Bangladesh exports live FWME to more than 15 countries with high demand in China, Thailand, Japan, Malaysia, South Korea, Taiwan, Hong Kong, and Singapore (Hasan et al., 2012). In 2016–2017, Bangladesh earned 25.37 million USD by exporting almost 1.3 million metric tons of FWME, contributing around 5% and 20% to total export value and export quantity of fish and fishery products, respectively (DoF, 2017). The tribal people believe this fish has therapeutic properties. Traditionally, it is used to treat various ailments, viz. weakness, asthma, anemia, hemorrhoids, and diabetes (Barman et al., 2013). To treat hemorrhoids, the dried head of FWME is cooked along with *Gracinia peduculata* and consumed (Acharyya & Sharma, 2004). Curry prepared by cooking the flesh of FWME along with certain herbs reportedly cures anemia, piles, and diabetes.

Aquatic food product plays a valuable role in providing nutrients necessary for diverse and healthy diets (Qara & Najafi, 2018). Assuring both the quality and safety of a aquafood will be a major challenge faced by humans in this new century. Cooking is inevitable for every animal food commodity, which effects its impacts on nutritional properties due to heat treatment. Food items like fish and meat become edible and more digestible after cooking. However, different types of heat treatment in cooking lead to undesirable modifications in food, like reduced nutritional values due to alternations in the components of protein fraction and lipid oxidation (Uran & Gokoglu, 2014). Therefore, the effects of cooking procedures on the quality of the final product are praiseworthy of investigation and interesting (Shi et al., 2020). In this study, different cooking methods were selected because there is great variations in the mechanisms of food heating. Thus, the effects of cooking methods have different effects on the nutritional properties of cooked products. For example, in microwave cooking, the alternating electric field provokes the rotation of dipole molecules in food (e.g., water), resulting in friction of heats in the food. Thus, it is a very fast thermal process in which the heating temperature is limited by the boiling point of the water molecule. Heating is applied in cooking, and different popular cooking processes such as grilling, boiling, frying, and microwaving are used to food to increase flavor and taste, deactivate microorganisms and pathogens, and extend shelf life (Bognar, 1998). The occurrence of changes in thermally processed cooked foods is protein denaturation, lipid oxidation, and development of Maillard reaction products. Lipid oxidation is common in thermal‐based cooking, which not only tremendously compromises food quality but also generates injurious and harmful compounds (Khalil & Mansour, 1998). In fish and fishery products, polyunsaturated fatty acids (PUFA) are considered highly prone to oxidation during heating and processing from cooking treatments. Therefore, an appropriate cooking method is essential to preserve maximum nutritional value such as proximate composition, amino acids, vitamins, fatty acids, and minerals (Momenzadeh et al., 2017).

Many reports are available on the nutrients intake from fish, most of which use data obtained from raw fish. However, so far, we know there is no report on nutritional status changes to FWME muscles due to thermal‐based processing. The fish species and the cooking methods are the determinant factors for nutritional uptake from consuming fish products. Preserving nutrients in fish is a major consumer demand related to preparation for the table. So far, the processors and the consumers have no information on how best to cook FWME muscle to maximize nutritional quality. Therefore, it is essential to measure the nutrients content in FWME muscle cooked according to popularly used domestic cooking practices. Also, there is no report on the minerals, heavy metal contents, and amino acid composition of FWME muscle. Cooking affects proximate composition, fatty acid and nutritional composition, minerals, and heavy metals concentration of fish muscle. It is important to evaluate the nutritional quality of FWME muscle processed by different types of cooking to provide information to the consumers about nutritional uptake. Therefore, this study aims to evaluate the proximate composition, nutritional parameters such as fatty acid composition, amino acid composition, minerals, heavy metal contents, and health risk assessment in raw and cooked FWME muscle.

## 2 MATERIALS AND METHODS

### 2.1 Collection and preparation of samples

Live FWMEs (82.50 ± 2.5 cm, 526 ± 32 g, *n* = 20) were collected from the Gabokhali Fish Farm, Monirampur, Jashore, Bangladesh, and transported to the laboratory. The fishes were sacrificed by beheading, and skins were removed. Then the fishes were cut into small pieces, approximately 4.0 ± 0.5 cm length, and each piece was 25.40 ± 2.45 g in weight. The chunks were washed with deionized cold water (4°C) and dewatered by keeping on a perforated tray for 20 min at room temperature (24°C).

### 2.2 Cooking the FWME muscles

Four commonly used popular cooking methods, grilling, boiling, frying, and microwaving, were used. The cooking temperature and times followed preliminary studies completed by a testing panel consisting of 10 expert members. After testing numerous combinations of cooking temperatures and times, the cooking conditions were suggested based on FWME muscle texture and tenderness (Feng et al., 2020). The muscle core temperature was measured immediately after cooking with a thermometer (Model: 104‐IR, Testo Instrument).

#### 2.2.1 Grilling

Grilling was performed using an electrically supported griller (Walton, Model: WG20 GL, Dhaka, Bangladesh) at 180°C for 20 min. The mean core temperature of the muscle was recorded at 80.5 ± 2.5°C.

#### 2.2.2 Boiling

Boiling of FWME muscle was done following the procedure of Armesto et al., (2019). FWME muscle was added to boiling water (FWME muscle: Water = 1:10) in a nonstick stainless steel pan (Model: Deep 26 Cm Ultra W/Glass Lid & Induction—Black, Prestige) and boiled at approximately 99°C for 10 min. The mean core temperature of immediately boiled fish was 89 ± 1.0°C.

#### 2.2.3 Frying

Freshwater mud eel muscle was fried using a nonstick aluminum fried pan (Model: Deep 26 Cm Ultra W/Glass Lid & Induction—Black, Prestige) at approximately 225°C for 5 min. The mean core temperature immediately after frying was recorded at 100 ± 2.5°C.

#### 2.2.4 Microwaving

Microwave‐baked of FWME muscle was done following the process of Nieva‐Echevarría et al. (2018) with slight modifications. Microwave‐baked FWME muscle was prepared using a domestic microwave oven (Walton, Model: WG30ESLR, Dhaka, Bangladesh) at 2,450 MHz for 10 min) containing FWME muscle was placed in a porcelain dish. Cooking was accomplished at 900 W for 5 min. The mean core temperature of the microwaved muscle immediately after cooking was 94.4 ± 1.2°C.

After cooking, all the cooked FWME muscle samples were cooled and the bones and skins were removed manually. Then the samples were homogenized in a high‐capacity food blender equipped with a stainless steel blade. Raw FWME muscle was also homogenized similarly to obtain the control sample. All the homogenized samples were stored in airtight polythene at −60°C for further analysis.

### 2.3 Analysis of proximate composition

The moisture, protein, and ash content of raw and cooked samples were determined according to the Association of Official Analytical Chemists (AOAC, 2010). Lipid content was determined by conventional Soxhlet extraction method using *n*‐hexane as an extracting solvent according to Ahmed et al. (2017). Carbohydrate content of raw and cooked FWME muscle was determined indirectly by substituting moisture, protein, and ash content from a hundred.

### 2.4 Analysis of amino acid composition

The amino acid content of raw and cooked FWME muscles was determined using an amino acid analyzer (LA 8080, Hitachi) equipped with a Hitachi high‐performance cation‐exchange column at 57°C column temperature. The sample was pretreated as‐ 0.5 g muscle was added with 25 ml of 6 N HCl in a glass tube. The tube was heated at 110°C for 24 hr, keeping in a heated sand bath. Then, the solid was dried by evaporating HCl and diluted with 6 ml distilled water and filtered by a 0.45 µm syringe filter.

### 2.5 Analysis of lipid profile

The fatty acid composition of raw and cooked FWME oils (previously extracted) was analyzed by using a 6,890 model gas chromatograph (Agilent Technologies) equipped with a fused silica capillary column (100 m length, 0.25 mm internal diameter, and 0.2 µm film) (Supelco). Fatty acid methyl esters were prepared following the method of the American Oil Chemists Society (AOAC, 2010). The oven temperature of gas chromatograph was programmed to arrive at 130°C in 3 min, later increased at a rate of 4°C/ min up to 240°C, and then soaked for 10 min. The injector and detector temperature were retained at 250°C. Standards of fatty acid methyl esters (Supelco^R^ 37 Component FAME Mix) were used for identifying the fatty acid methyl esters and quantification was done by obtained peak area (%).

### 2.6 Determination of minerals and heavy metals

Minerals and heavy metals content in raw and cooked FWME muscles were determined by using an ICP‐OES optima 2000 DV (Perkin Elmer) equipped with winLab32 software. The instrument's operational conditions were maintained as described previously (Kumaravel & Alagusundaram, 2014) with some modifications. Samples were prepared by a two‐step method. In the first step, samples were kept at 550°C overnight and were wholly converted to ashes. In the second step, 10 ml of digestion solution (a mixture of HNO_3_ and H_2_O_2_; 4:1) was mixed with 0.5 g ashed sample in a glass tube and heated at 200°C until the complete digestion of organic matter. This was indicated by the translucent and ending brownish smoke. Then, the tubes were cooled at room temperature and the digested samples were placed in a 50 ml volumetric flask accomplishing ash‐free filter paper (Whatman No. 1, Kent, England). Deionized distilled water was added to make the volume of the solution 50 ml and kept cool (4°C) until analysis. The minerals and heavy metals concentrations were measured in mg/100 g muscle wet weight basis.

### 2.7 Target hazard quotient (THQ)

Target hazard quotients (THQ) are an estimation of the noncarcinogenic risk level due to heavy metals exposure. THQ was calculated by using the following equation (Chien et al., 2002; USEPA, 2000):

$$\text{THQ}=\frac{\text{EF}\times \text{ED}\times \text{FIR}\times \text{Cf}\times \text{MC}}{\text{BW}\times \text{ATn}\times \text{RfD}}\times 10^{‐3}$$

where EF is the exposure frequency (350 days/year), ED is the exposure duration (30 years) for noncancer risk, as used by, FIR is the fish ingestion rate (49.5 g/day) (BBS, 2011), Cf is the conversion factor (00.208) to convert fresh weight (F_W_) to dry weight (D_W_) by considering 79% of moisture content in fish, MC is the heavy metal concentration in fish (mg/kg dry weight), BW is the average adult body weight (70 kg), and ATn is the average exposure time for noncarcinogens (EF × ED) (365 days/year × number of exposure years, assuming 30 years). RfD is the oral reference dose (mg/kg‐day) of individual metal, and RfD was used for analysis according to US EPA and previous studies (USEPA, 2020; Yi et al., 2017). A THQ value <1 indicates the exposed population is unlikely to experience adverse health hazards during a person's lifetime. In contrast, a THQ value equal to or greater than 1 indicates potential health risk (Wang et al., 2005), and related protective associated protective measures and interventions are necessary.

#### 2.7.1 Hazard index

The hazard index (HI) was expressed as the sum of the individual metal THQ values (USEPA, 2020).

HI=THQ(As)+THQ(Cd)+THQ(Cr)+THQ(Se)

where HI < 1 is safe, and HI > 1 is hazardous. THQ (As), THQ (Cd), THQ (Cr), and THQ (Se) is the target hazard quotient for As, Cd, Cr, and Se intake, respectively.

#### 2.7.2 Target cancer risk

Target cancer risk (TR) was used to indicate an incremental probability of an individual for developing cancer over a lifetime due to exposure to a potential carcinogen. The method to estimate TR is also provided in USEPA Region III Risk‐Based Concentration Table (USEPA, 2020). The model for estimating TR was shown as follows:

$$\text{TR}=\frac{\text{EF}\times \text{ED}\times \text{FIR}\times \text{Cf}\times \text{MC}\times \text{CPSo}}{\text{BW}\times \text{ATc}}\times 10^{‐3}$$

where CPSo is the carcinogenic potency slope for oral route (mg/kg bw/day), and ATc is the averaging time of carcinogens (365 days/year for 70 years), as used by USEPA (2020). Since CPSo values were known for As, Pb, and Cr, the TR values were calculated for in taking those metals.

### 2.8 Statistical analyses

Values are presented as means ± standard deviations of triple determinations. The data were analyzed by one‐way analysis of variance (ANOVA) using SPSS 20.0 (SPSS Inc.). Differences between the means were determined by Duncan's Multiple Range Tests (DMRT) and *p* < .05 was regarded as significant.

## 3 RESULTS AND DISCUSSION

### 3.1 Proximate composition

**Figure 1:**
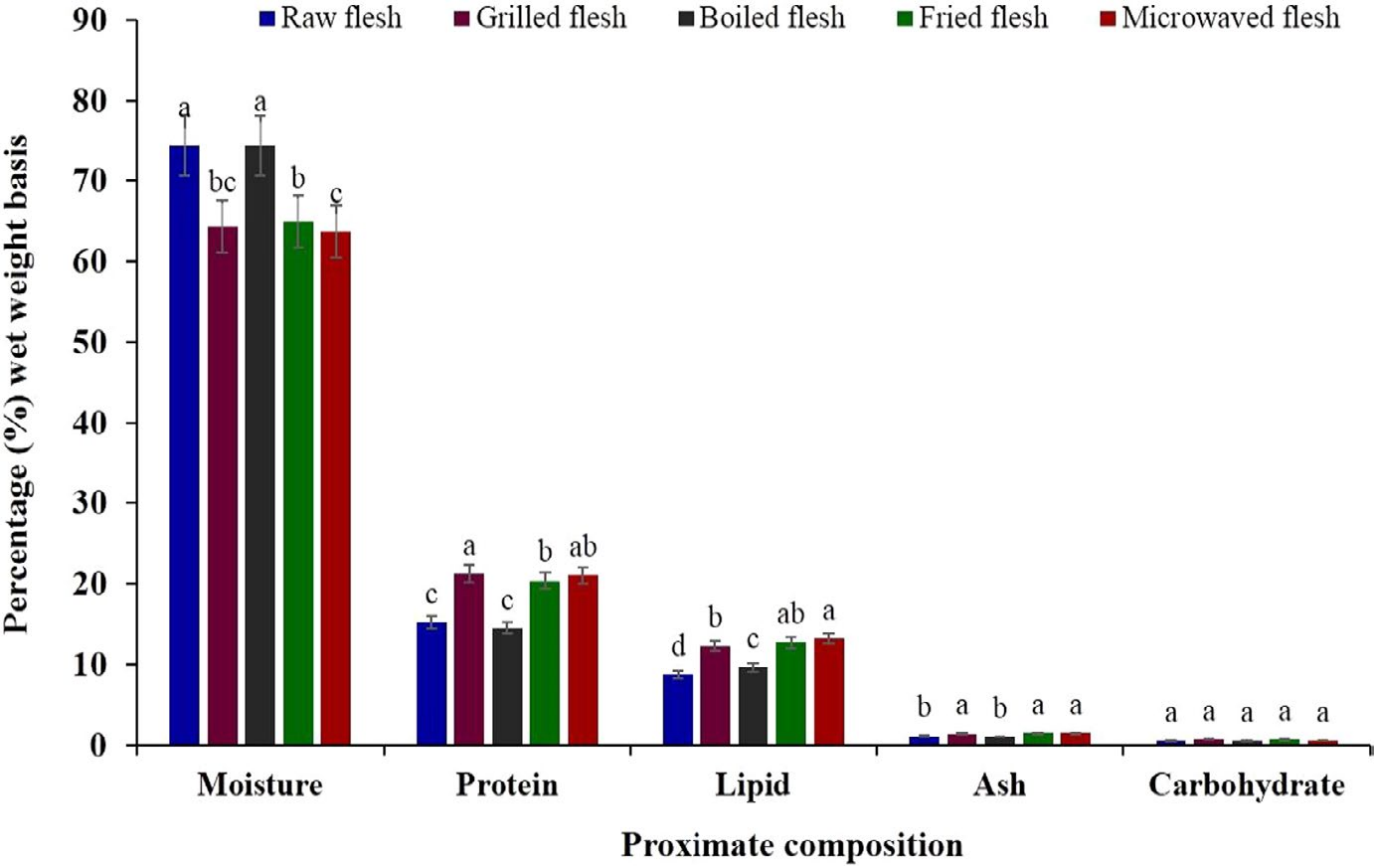
Proximate composition of raw and cooked FWME muscles. Different small letters on each bar indicate significant (*p* ≤ .05) differences. FWME, freshwater mud eel

The proximate composition, that is, moisture, protein, lipid, ash, and carbohydrate contents of raw and cooked FWME muscles are shown in Figure 1. The moisture content of raw FWME muscle was 74.45%, which decreased in all cooking methods except boiling. The moisture content of raw FWME muscle showed similarity with the previous report (Rana et al., 2019). However, in an earlier report, the moisture content of raw male and female FWME muscles was 81.25% and 80.71%, respectively (Faruque et al., 2019). The increment of moisture content in boiled FWME muscle might be because boiling was accomplished by a wet processing method. The moisture content in other cooking methods was reduced due to the removal of water in heat processing (Gokoglu et al., 2004). The lowest moisture content was observed in microwaved FWME muscle. Similar findings in moisture contents in cooked fish muscles were reported in previous reports (Tokur, 2007; Weber et al., 2008; Zhang et al., 2013).

The protein content in fresh and cooked FWME muscles varied between 14.49% and 21.28%. The protein content of raw FWME muscle was found 15.23%, which showed similarity with the previous report (Faruque et al., 2019). Among the cooked FWME muscles, the boiled sample showed the lowest protein content, which was found similar to the raw muscle. The reduction of protein content in boiled FWME muscle might be due to damage to the protein during boiling. The highest protein content was found in grilled and microwaved FWME muscles followed by fried ones. The protein content was increased due to the lower moisture content in cooked FWME muscles, and a similar result was reported by Weber et al., (2008). Fish is regarded as a high protein source when its protein content is more than 15%; therefore, FWME is considered a high protein fish. Based on dry weight, the lipid content of grilled, fried, and microwaved FWME muscles was increased significantly (*p* ≤ .05) compared to raw FWME muscle. The frying was done without cooking oil, so the loss of water was the sole contributor to the increment of lipid content (Hosseini et al., 2014). The lowest ash content was in the boiled FWME muscle followed by raw, grilled, fried, and microwaved FWME muscles. The mineral contents from the FWME muscles were leached out due to boiling process. The ash content of FWME muscles in other cooking methods showed higher values compared to raw FWME muscle. Raw and boiled FWME muscles contained a higher quantity of moisture compared to cooked FWME muscle. There is an inverse relationship between the moisture and lipid content in raw and cooked muscles (Hosseini et al., 2014). The increase of protein, lipid, and ash content in cooked muscles was due to loss of moisture during cooking (Ersoy & Özeren, 2009; Marimuthu et al., 2012).

### 3.2 Fatty acid composition

**Figure 2:**
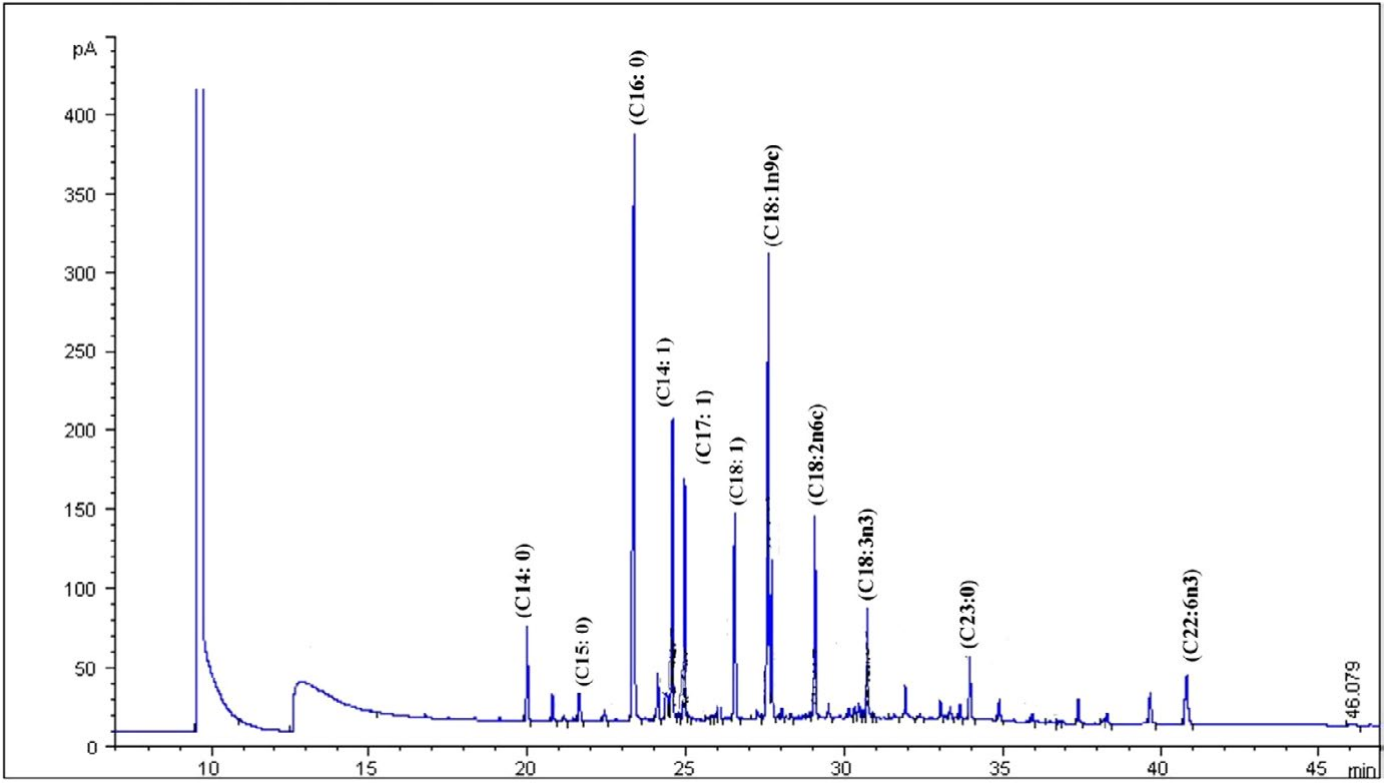
Gas chromatogram of raw FWME muscle with major fatty acids peaks. FWME, freshwater mud eel

The fatty acid profiles of raw and cooked FWME muscles are shown in Table 1, and the gas chromatogram of raw FWME muscle with major fatty acids peaks is presented in Figure 2. Twenty‐two fatty acids were detected in the oil of FWME muscle. The most abundant fatty acids were palmitic acid (26.51%), followed by oleic acid, palmitoleic acid, linoleic acid, stearic acid, and linolenic acid. There was a substantial amount of docosahexaenoic acid in the raw FWME muscle (5.06%). Fish is a significant dietary source of ω‐3 PUFAs, particularly EPA and DHA, and there are studies regarding the beneficial effects of their intake for coronary diseases. The total unsaturated fatty acids content in raw FWME muscle was 58.29%, which was higher than the total saturated fatty acids (SFAs). The results of fatty acid contents were in good agreement with the previous report (Rana et al., 2019). The cooking methods such as grilling, boiling, frying, and microwaving marginally affected the fatty acid composition of FWME muscles. The cooking procedures slightly increased the SFA contents from 41.71% to 42.84%, 46.07%, 43.27%, and 43.07% in grilled, boiled, fried, and microwaved FWME muscles, respectively. The increment in SFAs might be due to the decrement of ∑ω‐3 PUFAs contents in FWME muscles. The ∑ω‐3PUFAs in raw FWME muscle was found 13.70%, which was reduced to 10.95%, 7.54%, 11.11%, and 11.11% in grilled, boiled, fried, and microwaved FWME muscles, respectively. Similar results of increasing the SFAs content and decreasing ∑ω‐3PUFAs in fish muscles due to various cooking treatments were reported (Flaskerud et al., 2017; Zhang et al., 2013). Eicosapentaenoic acid, considered as one of the most important fatty acids in fish oil, was found in very poor quantities in raw and cooked FWME muscles, ranging from 0.53% to 0.79%. The docosahexaenoic acid content was 5.06% in raw FWME muscle whereas it was reduced significantly in boiled FWME muscle (2.71%). Faruque et al. (2019) reported the combined eicosapentaenoic acid and docosahexaenoic acid content in the male FWME muscle was 5.04%, which supports the present study's results. The reduction of docosahexaenoic acid, including ∑ω‐3 PUFAs might be due to the instability of docosahexaenoic acid and ω‐3 PUFAs in moist heat. The double bonds in PUFAs are highly prone to oxidation at high temperatures in the presence of moisture. Lipid oxidation is a significant problem in cooking, which reduces product quality, leading to an undesirable flavor, color, and an odor that affects the product's shelf life (Supawong et al., 2019). The results of fatty acids composition and its changes due to cooking showed good agreement with the previous reports (Bastías et al., 2017; Koubaa et al., 2012; Zhang et al., 2013).

**Table 1:** Fatty acids composition of raw and cooked FWME muscles

Fatty acids composition	Raw	Grilled	Boiled	Fried	Microwaved
Myristic acid (C14:0)	3.0 ± 0.36^a^	3.09 ± 0.2 ^a^	3.39 ± 0.31^a^	2.76 ± 0.26^b^	2.95 ± 0.23^a^
Pentadecanoic acid (C15:0)	1.01 ± 0.06^a^	1.01 ± 0.10^a^	1.11 ± 0.07^a^	0.88 ± 0.04^b^	0.16 ± 0.02^c^
Palmitic acid (C16:0)	26.51 ± 2.45	26.74 ± 2.84	29.70 ± 1.89	27.53 ± 2.49	27.42 ± 2.02
Palmitoleic acid (C16:1)	10.10 ± 0.91	10.32 ± 1.04	11.23 ± 0.85	9.50 ± 1.04	10.24 ± 0.58
Heptadecanoic acid (C17:1)	1.13 ± 0.30^a^	1.24 ± 0.21^a^	1.36 ± 0.31^a^	0.17 ± 0.07^b^	1.12 ± 0.11^a^
Stearic acid (C18:0)	7.45 ± 0.74	7.95 ± 0.89	8.92 ± 0.50	8.10 ± 0.27	8.80 ± 0.59
Elaidic acid (C18:1n9t)	0.46 ± 0.08^b^	0.48 ± 0.59^b^	0.53 ± 0.01^b^	1.91 ± 0.28^a^	0.44 ± 0.71^b^
Oleic acid (C18:1n9C)	17.77 ± 1.68	19.14 ± 2.90	21.09 ± 2.69	18.65 ± 2.08	19.26 ± 1.43
Linoleic acid (C18:2n6c)	8.51 ± 0.85^a^	7.86 ± 0.58^a^	7.00 ± 0.85^ab^	7.96 ± 0.93^a^	8.97 ± 0.74^a^
Arachidic acid (C20:0)	0.44 ± 0.08	0.43 ± 0.05	0.46 ± 0.08	0.76 ± 0.03	0.52 ± 0.06
r‐Linoleic acid (C18:3n6)	0.79 ± 0.10^b^	0.52 ± 0.07^c^	0.43 ± 0.05^c^	0.99 ± 0.08^a^	0.60 ± 0.04^c^
Eicosenoic acid (C20:1)	0.88 ± 0.04	0.79 ± 0.03	0.87 ± 0.08	1.63 ± 0.17	0.97 ± 0.07
Linolenic acid (C18:3n3)	6.91 ± 0.98^a^	4.88 ± 0.63^a^	3.76 ± 0.56^ab^	4.67 ± 0.48^a^	5.34 ± 0.45^a^
Eicosadienoic acid (C20:2)	1.53 ± 0.24	1.60 ± 0.15	1.60 ± 0.20	1.70 ± 0.12	1.64 ± 0.21
Eicosatrienoic acid (C20:3n6)	1.08 ± 0.05^a^	0.96 ± 0.10^a^	0.73 ± 0.08^ab^	1.08 ± 0.06^a^	1.10 ± 0.12^a^
Euric acid (C22:1n9)	0.38 ± 0.05	0.43 ± 0.02	0.46 ± 0.04	0.44 ± 0.03	0.47 ± 0.06
Eicosatrienoic acid (C20:3n3)	0.96 ± 0.16^a^	0.73 ± 0.10^a^	0.54 ± 0.07^b^	0.75 ± 0.08^a^	0.77 ± 0.05^a^
Tricosanoic acid (C23:0)	3.29 ± 0.25^a^	3.62 ± 0.31^a^	2.49 ± 0.17^b^	3.24 ± 0.38^a^	3.22 ± 0.31^a^
Docosadienoic acid (C22:2)	1.09 ± 0.06^b^	1.23 ± 0.14^a^	0.89 ± 0.09^b^	1.03 ± 0.06^b^	0.91 ± 0.07^b^
Eicosapentanoic acid (C20:5n3)	0.77 ± 0.11^a^	0.79 ± 0.09^a^	0.53 ± 0.06^b^	0.73 ± 0.10^a^	0.77 ± 0.10^a^
Nervonic acid (C24:1)	1.50 ± 0.18^a^	1.65 ± 0.11^a^	0.22 ± 0.05^b^	0.14 ± 0.01^b^	0.11 ± 0.01^b^
Docosahexanoic acid (C22:6n3)	5.06 ± 0.31^a^	4.55 ± 0.17^a^	2.71 ± 0.23^b^	4.96 ± 0.31^a^	4.23 ± 0.27^a^
∑SFAs	41.71	42.84	46.07	43.27	43.07
∑UFAs	58.29	57.16	53.93	56.73	56.93
∑ω‐3PUFAs	13.7	10.95	7.54	11.11	11.11

Note

Abbreviation: FWME, freshwater mud eel.

Values are presented as means ± standard deviation of triplicates. Different small letters on each row indicate significant (*p* ≤ .05) differences.

### 3.3 Amino acid composition

**Figure 3:**
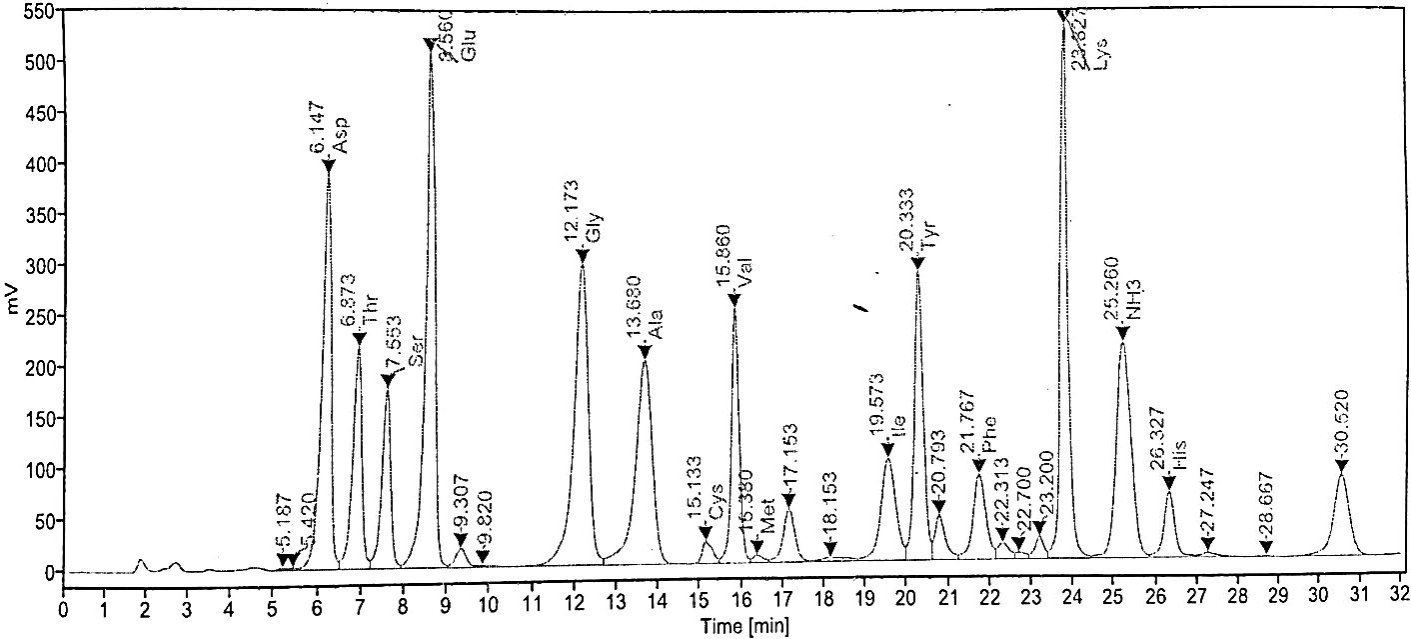
Amino acid chromatogram of raw FWME muscle. FWME, freshwater mud eel

**Figure 4:**
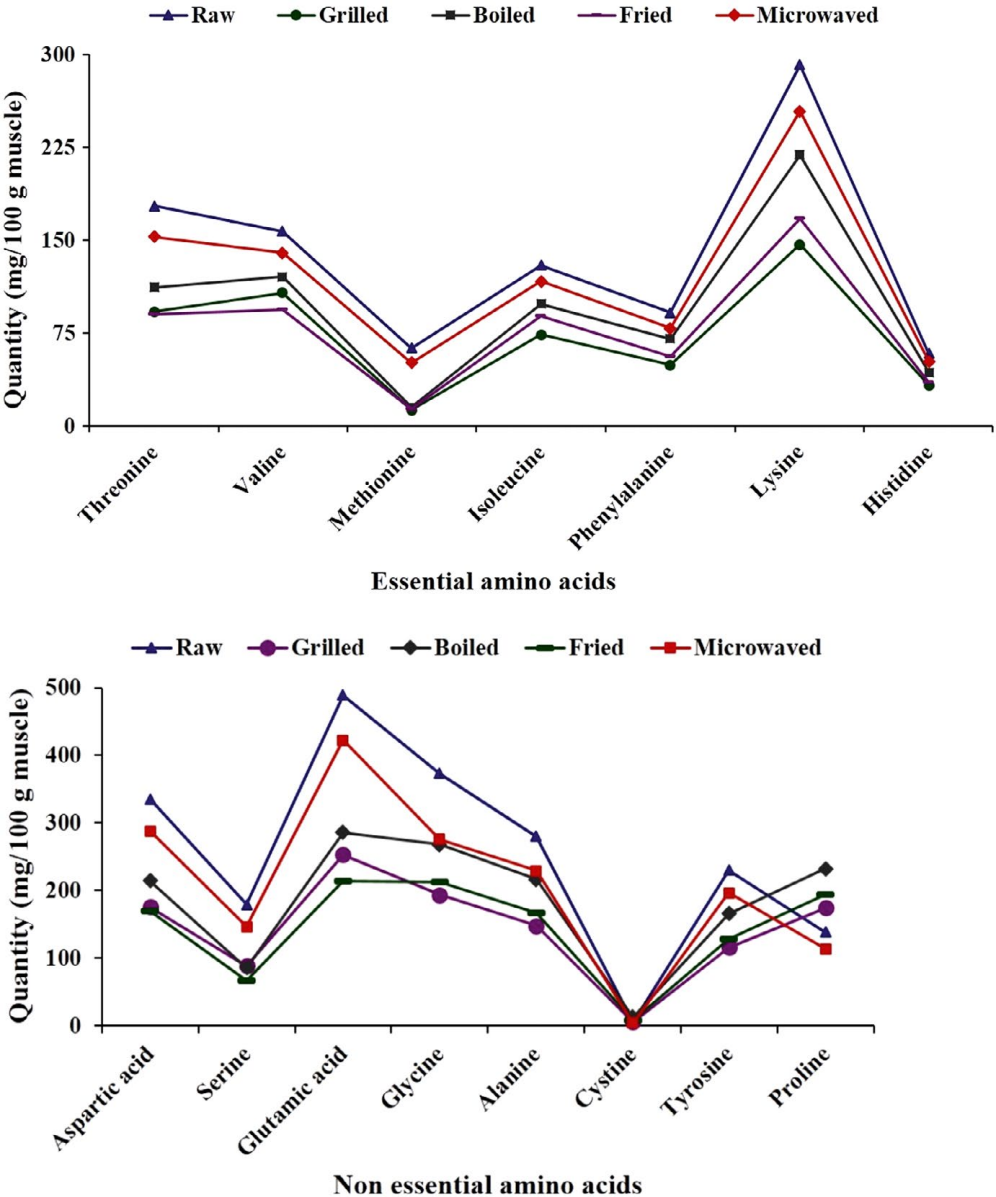
Amino acid contents of raw and cooked FWME muscles. FWME, freshwater mud eel

The amino acid chromatogram and amino acid profiles FWME muscles are shown in Figures 3 and 4, respectively. The essential amino acid composition in raw and cooked FWME muscles was varied due to cooking effects. Lysine was the highest amino acid in the raw FWME muscle (291.40 mg/100 g muscle) followed by threonine, valine, and isoleucine at the contents of 177.37, 156.77, and 129.43 mg/100 g muscle, respectively. Rana et al. (2019) analyzed the amino acids profile of FWME muscle and reported the highest content of amino acid was lysine. Lysine is an amino acid highly significant for a healthy diet and nutrition. This amino acid is limited in crops and cereal‐based products (Ghosh et al., 2012). So, FWME muscle might be an effective source for providing lysine to the cereal‐based diet consumers. A balanced diet comprising of essential amino acids is necessary for humans as they cannot synthesize essential amino acids (Asaduzzaman et al., 2018). The essential amino acid contents in both cooked and raw FWME muscles were higher compared to the suggested requirement by FAO/WHO (1995). In all the cooked FWME muscles, the amino acid contents were lower than the raw FWME muscle. This might be due to the heat sensitivity of amino acids. The amount of some essential amino acids were reduced in thermal‐based fish muscle processing (Asaduzzaman et al., 2018; Kim et al., 2013).

The nonessential amino acid contents in raw and cooked FWME muscles is shown in Figure 3. Among the nonessential amino acids in raw FWME muscle, glutamic acid content was maximum (488.67 mg/100 g muscle) followed by glycine, aspartic acid, and alanine 373.25, 335.01, 279.98 mg/100 g muscle, respectively. Cysteine was found the minimum amount at 5.36 488.67 mg/ 100 g muscle. There was some extent of nonessential amino acid reduction in cooked FWME muscles. The reduction in nonessential amino acid content was varied depending on the cooking methods as the thermal treatment and extent varied in different cooking methods.

### 3.4 Mineral content

The mineral content in raw and cooked FWME muscles is presented in Table 2. There were six minerals detected in raw and cooked FWME muscles among which calcium content was the highest. Calcium content varied between 794.52 and 883.24 mg/100 g muscle in FWME muscles. The calcium content in cooked FWME muscle increased significantly compared to raw FWME muscle. This value was higher than that reported by some researchers; however, the increased calcium content in cooked samples showed similarity with some reports (Ersoy & Özeren, 2009; Karimian‐Khosroshahi et al., 2016; Lall, 1995). Calcium is highly potential in performing various crucial and biofunctional activities such as contraction of muscles, building strong bones and teeth, nervous impulse, regulating heartbeat, balancing cell fluid, oocyte activation, and so on (Pravina et al., 2013). The Na content in raw FWME muscle was 125.40 mg/100 g muscle, whereas it increased to 184.72, 134.42, 180.52, and 170.72 mg/100 g muscle in grilled, boiled, fried, and microwaved FWME muscles, respectively. The Na concentrations in cooked FWME muscles increased by the four cooking methods; the reason is unclear. It might be because of heat treatment and reduced moisture content in the cooked products. The sodium content was found lowest in boiled FWME muscle which might be due to high moisture and leaching out some sodium content during boiling. The variation in sodium content due to the cooking of fish muscles was reported (Musaiger & D'Souza, 2008).

**Table 2:** Minerals content (mg/100g muscle) of raw and cooked FWME muscles

Minerals	Raw	Grilled	Boiled	Fried	Microwaved
Na	125.40 ± 4.76^d^	184.72 ± 5.38^a^	134.42 ± 4.92^c^	180.52 ± 5.90^a^	170.72 ± 5.39^b^
Mg	45.02 ± 2.49^b^	56.90 ± 2.95^a^	59.32 ± 3.02^a^	41.92 ± 2.39^c^	50.90 ± 3.86^ab^
K	38.70 ± 1.30^c^	47.76 ± 2.67^a^	39.90 ± 1.49^c^	40.48 ± 1.84^c^	43.76 ± 2.01^b^
Ca	794.52 ± 8.83^b^	878.36 ± 9.36^a^	802.90 ± 7.37^b^	883.24 ± 8.37^a^	798.36 ± 7.30^b^
Fe	12.38 ± 1.56^b^	14.16 ± 2.32^b^	14.84 ± 1.80^b^	13.6 ± 1.74^b^	21.42 ± 2.78^a^
Zn	3.56 ± 0.71^a^	5.04 ± 0.82^a^	3.90 ± 0.35^a^	4.32 ± 0.70^a^	3.44 ± 0.51^a^

Note

Abbreviation: FWME, freshwater mud eel.

Values are presented as means ± standard deviation of triplicates. Different small letters on each row indicate significant (*p* ≤ .05) differences (*n* = 3).

The magnesium content in raw and cooked FWME muscles varied between 41.92 and 59.32 mg/100 g muscle. The lowest value of magnesium was found in the boiled FWME muscle. There was a similar magnesium content in the cooked fish muscle (Musaiger & D'Souza, 2008). The potassium content in raw FWME muscle was 38.70 mg/100 g muscle which increased slightly in cooked FWME muscles 47.76, 40.48, and 43.76 mg/100 g in grilled, fried, and microwaved muscle, respectively. The changes in potassium content in cooked FWME muscle showed similar findings in the previous report. Potassium content in FWME muscles was found lower compared to reference values of fish muscle (Gokoglu et al., 2004; Hosseini et al., 2014; Musaiger & D'Souza, 2008). The Fe content in all the cooked FWME muscles showed higher values than raw FWME muscle. This might be associated with the thermal interactions of iron in FWME muscle and evaporation of water during the cooking process (Joyce et al., 2016). The iron content in raw FWME muscle was 12.38 mg/100 g muscle, which was similar to some previous reports (Uran & Gokoglu, 2014; Wheaton & Lawson, 1985). The zinc content in raw and cooked FWME muscles varied between 5.04 and 3.44 mg/100 g muscle. There were no notable changes in zinc content due to the cooking effect. Fe and Zn are considered as necessary for the human body, but these might have a harmful effect on the human body at a higher dose (Singh et al., 2011). Moreover, Fe and Cu perform pro‐oxidative roles by catalyzing lipid oxidation since the activity of Cu is 10X higher than Fe. Essential minerals such as zinc, copper, iron, selenium, calcium, and manganese are highly beneficial for their biological functions in living organisms. Minerals are essential for the growth and development of organisms as they perform crucial physiological and metabolic activities in the living body (Abdulkarim et al., 2015). In this study, the mineral content in the cooked FWME was suitable for human consumption in aspects of mineral content. Cooking methods had significant effects on Na, Ca, Mg, Zn, and Cu contents in fish (Uran & Gokoglu, 2014). The mineral levels in some fish samples were affected by cooking methods (Ackurt, 1991; Salawu et al., 2005).

### 3.5 Changes in heavy metals content

**Figure 5:**
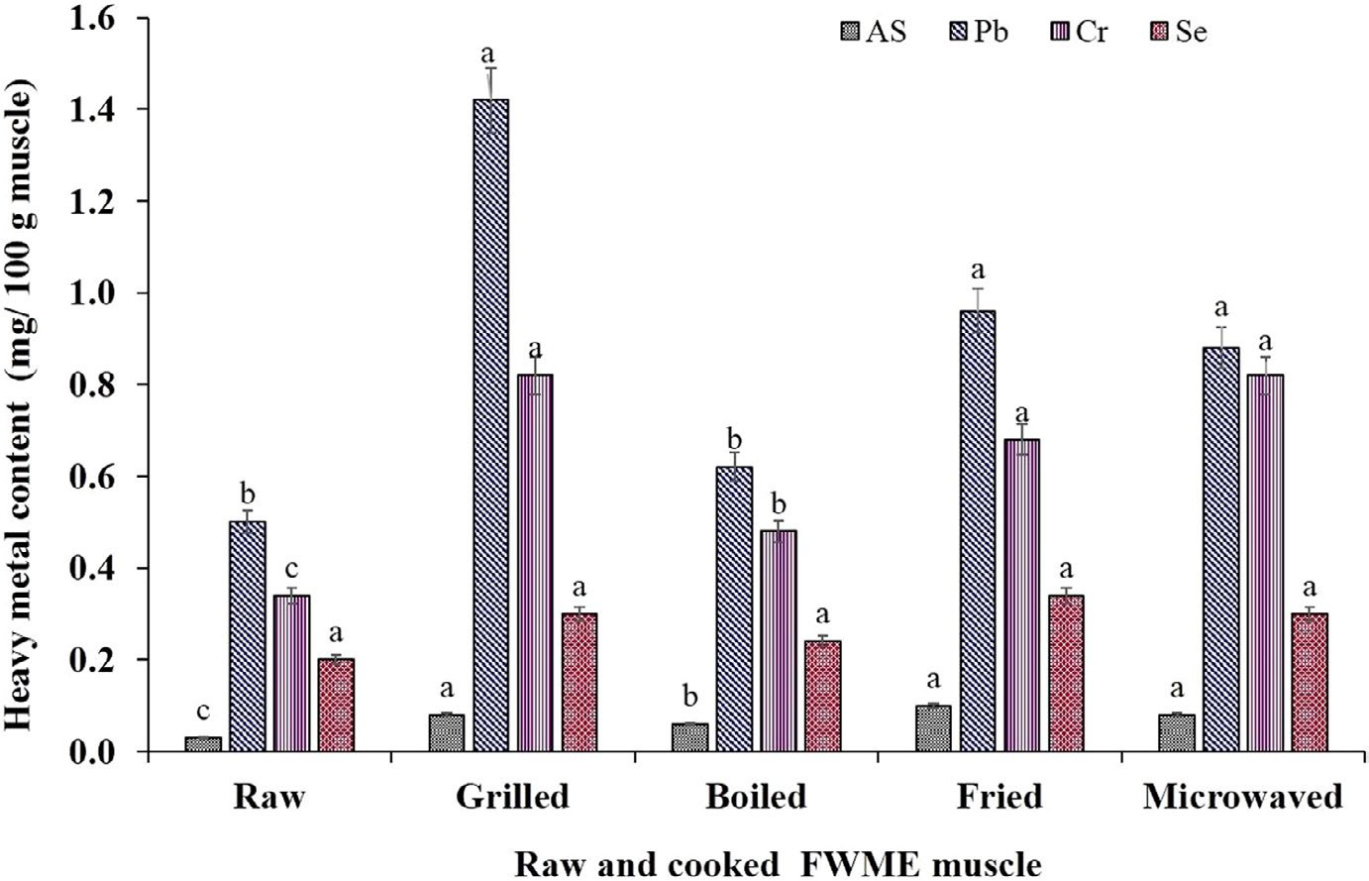
Heavy metals content (mg/100g muscle) of raw and cooked FWME muscles. Different small letters on each group of heavy metal indicate significant (*p* ≤ .05) differences. FWME, freshwater mud eel

The heavy metals content in raw and cooked FWME muscles are shown in Figure 5. The As concentration in raw FWME muscle was 0.03 mg/100 g muscle. The As concentration increased slightly in the cooked FWME muscles. In the cooked samples, the As concentration was 0.08, 0.06, 0.10, and 0.08 mg/100 g muscle in grilled, boiled, fried, and microwaved FWME muscles, respectively. The increment of total As in cooked FWME muscle is mostly due to loss of water during cooking. The result is in agreement with the previous report (Devesa et al., 2001). The As concentration of FWME muscles was lower than some fish samples reported by some other researchers (Ahmed et al., 2017; Ersoy et al., 2006). As contamination mostly occurs in aquatic organisms reared by underground or marine water. The FWME studied in the present study was reared by surface/ runoff water which rendered very low As level deposition in FWME muscle.

The Pb concentration in raw FWME muscle was found 0.50 mg/100 g muscle. In the cooked FWME muscles, the Pb concentration was found to be 1.42, 0.62, 0.96, and 0.88 mg/100 g muscle in grilled, boiled, fried, and microwaved samples, respectively. The Pb concentrations increased in cooked FWME products due to the reduction of water in the cooking process. The value of Pb content obtained in this study was higher than in some previous reports (Dugo et al., 2006; Ersoy et al., 2006). There were no significant differences in Pb content in grilled, fried, and microwaved samples; however, boiled samples showed lower values.

The Cr content in raw FWME muscle was found 0.34 mg/100 g muscle which increased to 0.82, 0.48, 0.68 and 0.82 mg/100 g in grilled, boiled, fried, and microwaved muscles, respectively. The value of Cr concentration in the FWME muscles was lower than sea bass fillet (Ersoy et al., 2006). The lowest Se concentration was found in raw FWME muscle whereas the highest concentration was in fried FWME muscle. However, there was no significant difference between the raw and cooked FWME muscles (*p* ≤ .05). There are some changes in the concentration of heavy metals due to the effects of cooking in FWME muscles, possibly because of moisture content in the final product and the transformation of heavy metals in different forms as a consequence of thermal effects (Devesa et al., 2001; van Elteren & Šlejkovec, 1997; Jorhem et al., 1994).

### 3.6 Health risk assessment

The exposure to heavy metals through the consumption of FWME could affect consumer health. Therefore, the health risk assessment is certainly needed for those consumers who eat FWME muscles daily.

#### 3.6.1 Target hazard quotient

Estimated THQ for As, Pb, Cr, and Se through the consumption of FWME muscle in different forms are shown in Table 3. According to USEPA, the acceptable value is 1 for THQ (USEPA, 2020). The THQ value of each heavy metal was less than 1, indicating that people would not experience potential carcinogenic health risks if they only intake one heavy metal individually (Heshmati et al., 2019) through the consumption of FWME muscle. The combined effects of all metals under consideration were higher than the acceptable limit of 1 for HI in grilled, fried, and microwaved FWME muscles. However, in the case of raw and boiled FWME muscles, the HI values were below the acceptable limit. The obtained values revealed that the excessive and long‐term intake of these fish especially grilled, fried, and microwaved muscles could result in potential chronic noncarcinogenic health risks.

**Table 3:** Target hazard quotients (THQ) for individual metals and their hazard index (HI) from consumption of raw and cooked freshwater mud eel (FWME) muscles

Heavy metals	Raw	Grilled	Boiled	Fried	Microwaved
AS	0.141 ± 0.01^d^	0.376 ± 0.02^b^	0.282 ± 0.02^c^	0.470 ± 0.04^a^	0.376 ± 0.03^b^
Pb	0.176 ± 0.01^d^	0.500 ± 0.03^a^	0.218 ± 0.01^c^	0.338 ± 0.02^b^	0.310 ± 0.02^b^
Cr	0.159 ± 0.01^c^	0.385 ± 0.05^a^	0.225 ± 0.02^b^	0.319 ± 0.03^a^	0.385 ± 0.03^a^
Se	0.056 ± 0.01^c^	0.084 ± 0.01^a^	0.067 ± 0.01^b^	0.095 ± 0.02^a^	0.084 ± 0.01^a^
HI	0.533	1.346	0.794	1.224	1.156

Note

Values are presented as means ± standard deviation of triplicates. Different small letters on each row indicate significant (*p* ≤ .05) differences (*n* = 3).

#### 3.6.2 Target cancer risk

The carcinogenic potency slope factor is available for As, Pb, and Cr. Inorganic As is classified as a known carcinogen (USEPA group A) and a Pb as probable carcinogen based on animal studies (USEPA group B2). Thus, the lifetime TR of As, Pb, and Cr for adults due to exposure from fish consumption is listed in Table 4. The TR values for As, Pb, and Cr ranged from 2.72E‐05 to 9.06E‐05, 0.26E‐05 to 0.73E‐05, and 10.20E‐05 to 24.70E‐05, respectively, in raw and cooked FWME muscles (Table 4). Generally, a cancer risk lower than 10^−6^ is negligible, a cancer risk above 10^−4^ is unacceptable, and risk between 10^−6^ and 10^−4^ is acceptable (USEPA, 2010). The carcinogenic risk for As and Pb was within the acceptable range in raw and cooked FWME muscles. In contrast, TR for Cr was higher than the unacceptable value in all samples indicating the risk of cancer due to exposure to Cr through the excessive consumption of fish over a prolonged period. Summarizing the present study, possible health risks to people due to heavy metal exposure through fish ingestion should not be overlooked. Also, other sources of metal exposures, such as consumption of other foodstuffs (e.g., groundwater, wheat, rice, pulses, meat, and egg) and dust inhalation, are not included in this study.

**Table 4:** Target cancer risk (TR) for heavy metals from consumption of raw and cooked freshwater mud eel (FWME) muscles

Heavy metals	Raw	Grilled	Boiled	Fried	Microwaved
AS	2.72E−05	7.25E−05	5.44E−05	9.06E−05	7.25E−05
Pb	0.26E−05	0.73E−05	0.32E−05	0.49E−05	0.45E−05
Cr	10.20E−05	24.70E−05	14.5E−05	20.5E−05	24.70E−05

## 4 CONCLUSION

This study provides information about the proximate composition and nutritional status of FWME muscles and the effects of cooking as well. The proximate composition changed due to the effects of cooking methods. Removal of moisture in grilling, frying, and microwaving methods increased protein content in cooked FWME muscles. The lipid content was increased in cooked samples except for boiled FWME muscle as it contained a similar quantity of moisture compared to the raw muscle. The FWME muscles were rich in ω‐3 PUFAs. Still, the boiling process decreased the content significantly, possibly because of the oxidation of long‐chain fatty acids in moist heat. Other fatty acids were marginally affected by different cooking methods. Lysine, a very important essential amino acid, was the highest in FWME muscles. The cooking methods slightly affected mineral contents in raw and cooked FWME muscles. However, the reasons for the increment are unclear. A very high level of Ca content, including some other minerals, made FWME muscles a recommended food for Ca‐ and minerals‐deficient people. The FWME muscles were found safe in health risk assessments regarding the contents of heavy metals.

## CONFLICT OF INTEREST

The authors declare no conflict of interest.

## ETHICAL APPROVAL

This study did not involve any animal or human testing.
